# Prophylactic noninvasive respiratory support in the immediate postoperative period after cardiac surgery - a systematic review and network meta-analysis

**DOI:** 10.1186/s12890-023-02525-1

**Published:** 2023-06-28

**Authors:** Xiaoyang Zhou, Jianneng Pan, Hua Wang, Zhaojun Xu, Long Zhao, Bixin Chen

**Affiliations:** 1 Department of Intensive Care Medicine, Ningbo No.2 Hospital, Ningbo, 315000 Zhejiang China; 2 Department of Cardiovascular Surgery, Ningbo No.2 Hospital, Ningbo, Ningbo, 315000 Zhejiang China

**Keywords:** Atelectasis, Cardiac surgery, Continuous positive airway pressure, High flow nasal cannula, Non-invasive ventilation, Postoperative pulmonary complications

## Abstract

**Background:**

Noninvasive respiratory support has been increasingly applied in the immediate postoperative period to prevent postoperative pulmonary complications (PPCs). However, the optimal approach remains uncertain. We sought to evaluate the comparative effectiveness of various noninvasive respiratory strategies used in the immediate postoperative period after cardiac surgery.

**Methods:**

We conducted a frequentist random-effect network meta-analysis (NMA) of randomized controlled trials (RCTs) comparing the prophylactic use of noninvasive ventilation (NIV), continuous positive airway pressure (CPAP), high flow nasal cannula (HFNC), or postoperative usual care (PUC) in the immediate postoperative period after cardiac surgery. Databases were systematically searched through September 28, 2022. Study selection, data extraction, and quality assessment were performed in duplicate. The primary outcome was the incidence of PPCs.

**Results:**

Sixteen RCTs enrolling 3011 patients were included. Compared with PUC, NIV significantly reduced the incidence of PPCs [relative risk (RR) 0.67, 95% confidence interval (CI): 0.49 to 0.93; absolute risk reduction (ARR) 7.6%, 95% CI: 1.6–11.8%; low certainty] and the incidence of atelectasis (RR 0.65, 95% CI: 0.45 to 0.93; ARR 19.3%, 95% CI: 3.9–30.4%; moderate certainty); however, prophylactic NIV was not associated with a decreased reintubation rate (RR 0.82, 95% CI: 0.29 to 2.34; low certainty) or reduced short-term mortality (RR 0.64, 95% CI: 0.16 to 2.52; very low certainty). As compared to PUC, the preventive use of CPAP (RR 0.85, 95% CI: 0.60 to 1.20; very low certainty) or HFNC (RR 0.74, 95% CI: 0.46 to 1.20; low certainty) had no significant beneficial effect on the incidence of PPCs, despite exhibiting a downward trend. Based on the surface under the cumulative ranking curve, the highest-ranked treatment for reducing the incidence of PPCs was NIV (83.0%), followed by HFNC (62.5%), CPAP (44.3%), and PUC (10.2%).

**Conclusions:**

Current evidence suggest that the prophylactic use of NIV in the immediate postoperative period is probably the most effective noninvasive respiratory approach to prevent PPCs in patients undergoing cardiac surgery. Given the overall low certainty of the evidence, further high-quality research is warranted to better understand the relative benefits of each noninvasive ventilatory support.

**Clinical Trial Registration:**

PROSPERO, https://www.crd.york.ac.uk/prospero/, registry number: CRD42022303904.

**Supplementary Information:**

The online version contains supplementary material available at 10.1186/s12890-023-02525-1.

## Introduction

Postoperative pulmonary complications (PPCs) represent a composite outcome of minor and major pulmonary complications that are commonly encountered in the postoperative period, with a reported prevalence of up to 30–50% in patients undergoing cardiothoracic surgery [1–3]. The high prevalence of PPCs in cardiac surgery partly attributes to the nature of the direct intrathoracic procedure. During the perioperative period of intrathoracic surgery, multiple risk factors contribute to the susceptibility to developing PPCs in such a population [4]. Of these, cardiopulmonary bypass is a critical predisposing factor that can precipitate lung inflammation and ischemia-reperfusion injury and finally cause substantial pulmonary compromise [4]. Alterations of chest wall structure and function further impair lung expansion and aggravate lung atelectasis. Additionally, potential diaphragm impairment during intrathoracic surgery can lead to impaired sputum expectoration and inspiratory muscle weakness. These adverse factors together induce unfavorable changes in respiratory pathophysiology [5], thereby resulting in PPCs and postoperative respiratory dysfunction.

Currently, PPCs are widely recognized as a leading cause of increased morbidity and mortality in cardiac surgery [3, 6]. It is thus essential to prevent PPCs in patients undergoing cardiac surgery, anticipating improved clinical outcomes. Over the past decade, noninvasive positive pressure ventilation (NPPV), including noninvasive ventilation (NIV, delivered as bilevel positive airway pressure) and continuous positive airway pressure (CPAP), and high flow nasal cannula (HFNC) have been increasingly utilized in the immediate postoperative period to prevent PPCs. Both NPPV and HFNC elicit a series of beneficial physiological effects on cardiovascular and respiratory function. By delivering a stable positive airway pressure at inspiration and expiration levels, NIV and CPAP can promote alveolar recruitment, prevent alveolar collapse, mitigate ventilation-perfusion mismatch, and reduce left ventricular pre- and afterload, and thus improve cardiorespiratory performance [7]. Apart from generating a low level of positive airway pressure, HFNC also washes out the upper airway dead space and facilitates the clearance of secretions [8–10]. Consequently, these noninvasive respiratory approaches are considered promising complementary treatments to postoperative usual care (PUC).

However, the optimal approach of noninvasive respiratory support used in the immediate postoperative period remains unknown. A recent meta-analysis suggested that the treatment with NPPV after cardiac surgery did not affect the incidence of PPCs [11], and two meta-analyses found conflicting results in terms of the length of in-hospital stay and reintubation with the prophylactic use of HFNC after cardiac/thoracic surgery [12, 13]. Although these conventional pairwise meta-analyses are informative, they cannot inform on the relative effect of indirectly compared approaches without head-to-head comparisons. As an alternative, network meta-analysis (NMA) can overcome this limitation and compare multiple treatments simultaneously in a single analysis by combining direct and indirect evidence [14]. Therefore, we conducted this NMA to assess the comparative effectiveness of various noninvasive respiratory methods used in the immediate postoperative period after cardiac surgery.

## Methods

This systematic review with NMA was reported following the Preferred Reporting Items for Systematic Reviews and Meta-Analyses (PRISMA) Extension statement for reporting network meta-analyses [15]. The study protocol was registered at the international prospective register of systematic reviews (PROSPERO; CRD42022303904). Institutional review board approval was not required due to the nature of the review article.

### Literature search and study selection

Two independent reviewers (JP and HW) systematically searched the PubMed, Embase, Web of Science, and Cochrane Controlled Clinical Trial Register to identify randomized controlled trials (RCTs) comparing the prophylactic use of NIV, CPAP, HFNC, or PUC in the immediate postoperative period in adult patients undergoing cardiac surgery. Electronic database searching was completed on January 23, 2022, and updated on September 28, 2022. The comprehensive search strategies are presented in Additional File 1. We also manually searched the bibliographies of previous publications to further identify relevant literature.

Duplicate records searched from each database were initially auto-filtered for deduplication. Then, the title and abstract of the remaining records were screened independently for eligibility by the same two reviewers (JP and HW). The full texts of all records deemed relevant were reviewed carefully. Disagreements between the two reviews were adjudicated by a discussion with a third reviewer (XZ). No restriction was applied to language or publication date. The excluded studies with associated reasons are listed in Additional File 1.

### Eligibility criteria

Candidate studies were screened in compliance with the following eligibility criteria: (1) Participants: adult patients (age greater than 18 years) who had undergone cardiac surgery, regardless of the urgency, and were successfully extubated; (2) Interventions and comparators: CPAP, NIV, HFNC, and PUC. All the noninvasive respiratory methods were initiated in the immediate period (no more than 6 h) following extubation after surgery for prophylactic purposes; (3) Outcomes: the interested outcomes included the incidence of PPCs, atelectasis, reintubation, short-term mortality, and lengths of intensive care unit (ICU) stay and in-hospital stay. Studies reporting on at least one of the above outcomes were included; (4) Study design: prospective RCT. We excluded those studies that met anyone of the following criteria: (1) Studies without randomized controlled design; (2) Studies conducted in patients who had developed postoperative respiratory failure; (3) Studies that did not report any outcome of interest; (4) Studies in which noninvasive respiratory support had been initiated before surgery, or was used for therapeutic or facilitative purposes [9], or was not used in the immediate postoperative period; (5) Studies conducted in other surgery types rather than cardiac surgery; (6) Studies with a sample size of less than 30; (7) Conference abstracts without a full text.

### Outcomes and definitions

The primary outcome was the incidence of PPCs, with PPCs being pre-defined as the composite of any of pneumonia, atelectasis, acute respiratory distress syndrome, or pulmonary aspiration [16], or defined by the authors in the original articles. The secondary outcomes included the incidence of atelectasis, reintubation rate, short-term mortality, and lengths of ICU stay and in-hospital stay. These secondary outcomes were defined by the authors in the included studies and measured at the longest time point reported up to 30 days. Short-term mortality was defined as death within 30 days after randomization. If studies reported various mortalities, the longest follow-up short-term mortality was included.

### Data extraction and risk of bias assessment

Two reviewers (LZ and ZX) independently extracted data from each included study, including the following contents: study and patient characteristics, intervention details, and outcome measures, with discrepancies being resolved by a third reviewer (BC). If necessary, the corresponding author would be contacted to clarify information as required; in reality, however, no author was contacted despite sending an inquiring email. The methodological quality of individual studies was assessed independently by the same two reviewers for the primary outcome using the Cochrane Collaboration’s risk of bias tool 2.0 [17]. We constructed a “traffic light” plot to illustrate the risk of bias assessment in each domain [18]. Studies with a low risk of bias in all domains were considered as overall low risk of bias. A panel of reviewers (LZ, BC, and ZX) participated in the discussion to reach a consensus.

### Statistical analysis

Initially, conventional pairwise meta-analyses with random-effects models were performed to obtain the direct effect estimates from head-to-head comparisons for all outcomes. A series of frequentist random-effects NMA was then conducted to derive the relative treatment estimates of all interventions, allowing for the expected substantial heterogeneities among the included studies. Categorical outcomes were summarized as relative risk (RR) and continuous data were presented as mean differences (MD), accompanied by corresponding 95% confidence intervals (CI). Additionally, we calculated the absolute treatment effect for the categorical outcomes using the assumed event rate across all trials in the PUC arm. Data syntheses were performed in Stata/SE 15.0 software (Stata-Corp, College Station, TX, USA) with the mvmeta, network, and network graphs packages. A two-sided *P* value < 0.05 was considered statistical significance.

Before synthesizing data, we comprehensively confirmed the transitivity, consistency, and homogeneity assumptions, which underlie the validity of NMA evidence [19, 20]. Network plots were constructed to describe the connectivity between different interventions. We evaluated the distribution of patients and study characteristics that might modify treatment effects to assess the transitivity across different comparisons. The coherence assumption in the entire network was assessed using a design-by-treatment interaction model (global test); the incoherence between direct and indirect effect estimates was evaluated using the side-splitting method (local test) [21]. We assessed the heterogeneities among included studies by calculating the Q test and the I^2^ statistic and visually inspecting the forest plots [22]. The hierarchy of various interventions was ranked by calculating the surface under the cumulative ranking curve (SUCRA) value for each outcome. The SUCRA value, ranging from 0 to 100%, represents the probability of treatment effectiveness ranking highest [23]. The presence of small-study effects for the primary outcome was evaluated by generating the comparison-adjusted funnel plot. If sufficient studies were included, we would conduct a sensitivity analysis to evaluate the effect of a potential effect modifier (age over 60 years or not) on the robustness of the NMA results.

### Assessment of certainty of evidence

The GRADE four-step approach was implemented to rate the certainty of evidence in each of the direct, indirect, and NMA estimates for each comparison [24]. Downgrading the certainty was based on the presence of risk of bias, inconsistency, indirectness, imprecision, incoherence, or publication bias [24]. Of note, imprecision assessment was only performed at the network level, but not at the level of the direct or indirect estimate [25]. We focused on the most-dominant first-order loop to rate the certainty of the indirect estimate, which was assigned the lowest rating in the contributing direct comparisons within the first-order loop. The higher certainty rating of the direct and indirect estimates was assigned as the certainty of the NMA estimate.

## Results

**Fig. 1:**
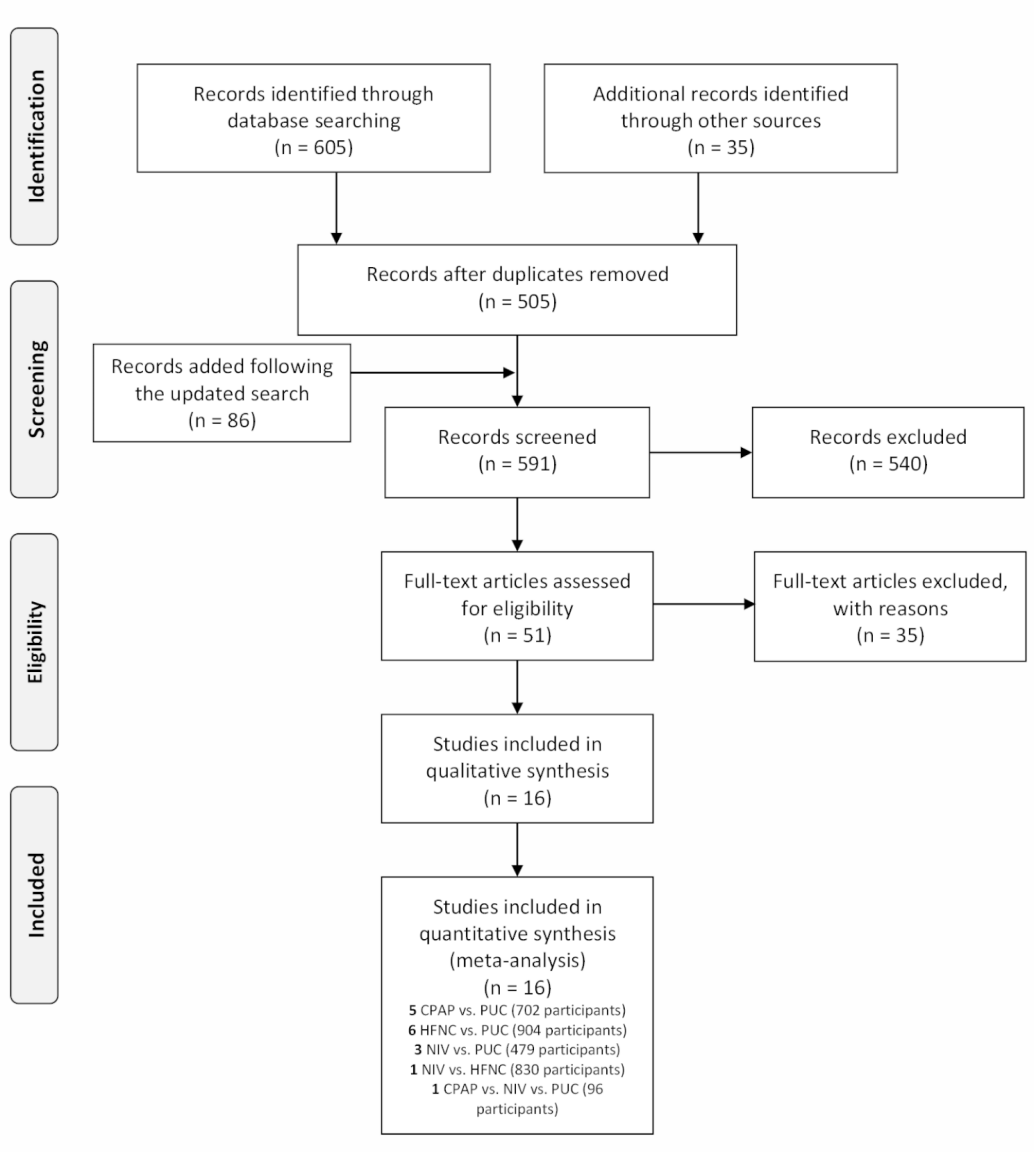
The PRISMA flowchart of study selection. CPAP continuous positive airway pressure; NIV noninvasive ventilation; HFNC high flow nasal cannula; PUC postoperative usual care

The initial search yielded a total of 640 records, and additional 86 citations were added following the updated search. After deduplication and exclusion of irrelevant records, 16 eligible RCTs [26–41] that enrolled 3011 participants were included in the quantitative analyses. Figure 1 depicts the PRISMA flowchart of study selection.

**Fig. 2:**
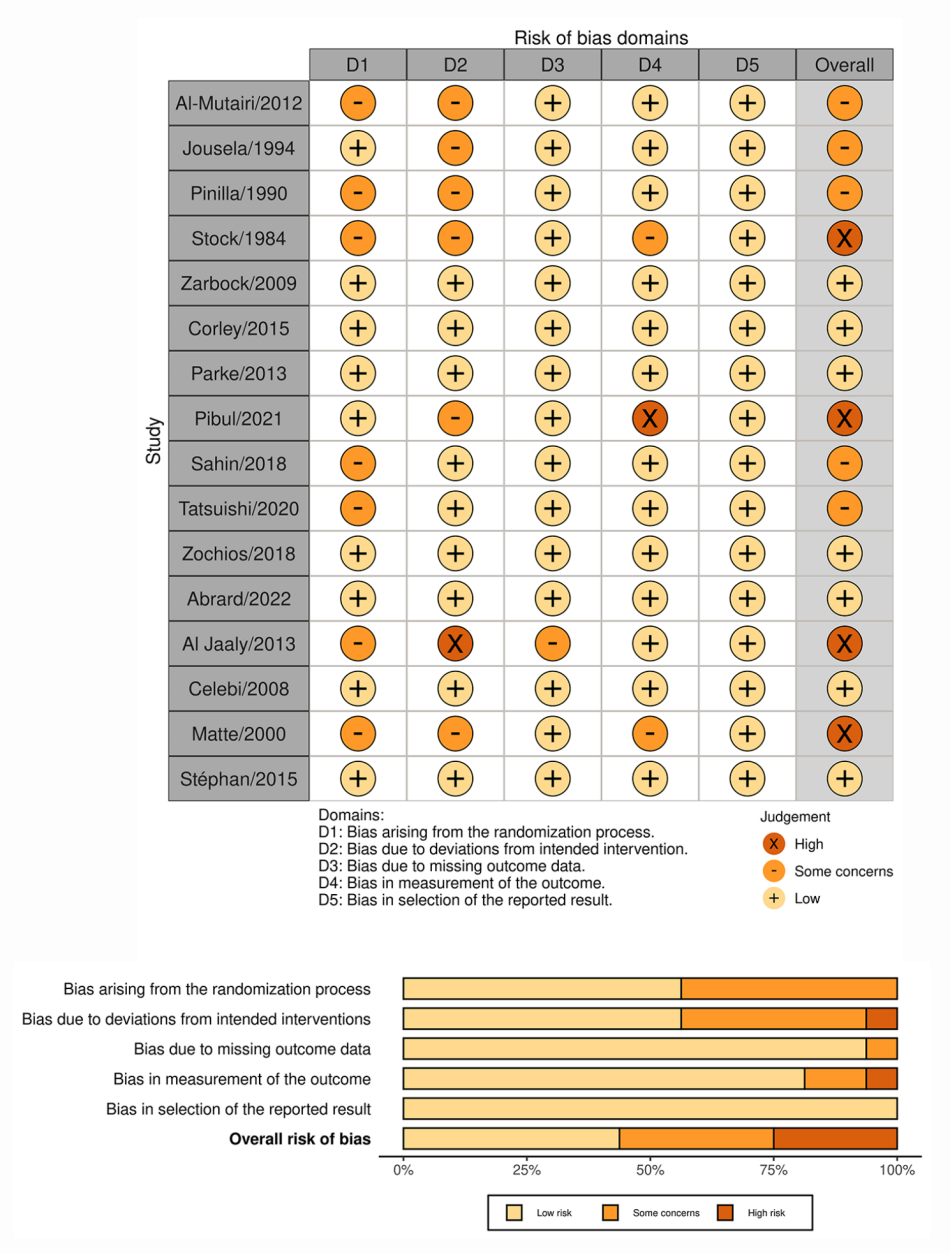
Assessment of risk of bias for included studies

The study characteristics and outcome measures are displayed in Table S1 and S2 (see Additional file 2), respectively. Although the distribution of the patient and study characteristics were not fully balanced among the included trials, the variation was not large enough to warrant concerns regarding intransitivity. Among the 16 included trials, the majority (81%) was single-center [26–29, 31–36, 38–40]; the sample sizes ranged from 30 to 830, and the mean age ranged from 53 to 69 years. Of the 16 included RCTs, 5 [26–30] compared CPAP with PUC, 6 [31–36] compared HFNC with PUC, 3 [37–39] compared NIV with PUC, 1 [41] compared NIV with HFNC, and the last one [40] was a 3-arm study comparing CPAP with NIV and with PUC. As shown in the “traffic light” plot (Figs. 2), 7 trials were judged as overall low risk of bias [30–32, 36, 37, 39, 41].

### Primary outcome and sensitivity analyses

**Fig. 3:**
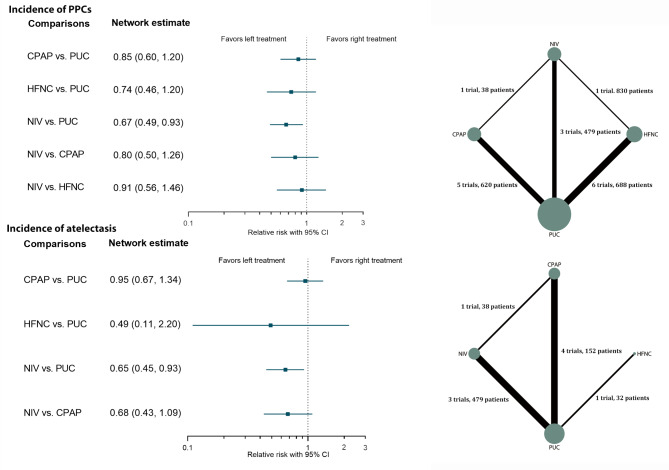
Forest plots of network meta-analysis for the incidence of PPCs and atelectasis. PPCs postoperative pulmonary complications; CPAP continuous positive airway pressure; NIV noninvasive ventilation; HFNC high flow nasal cannula; PUC postoperative usual care; CI confidence interval

**Fig. 4:**
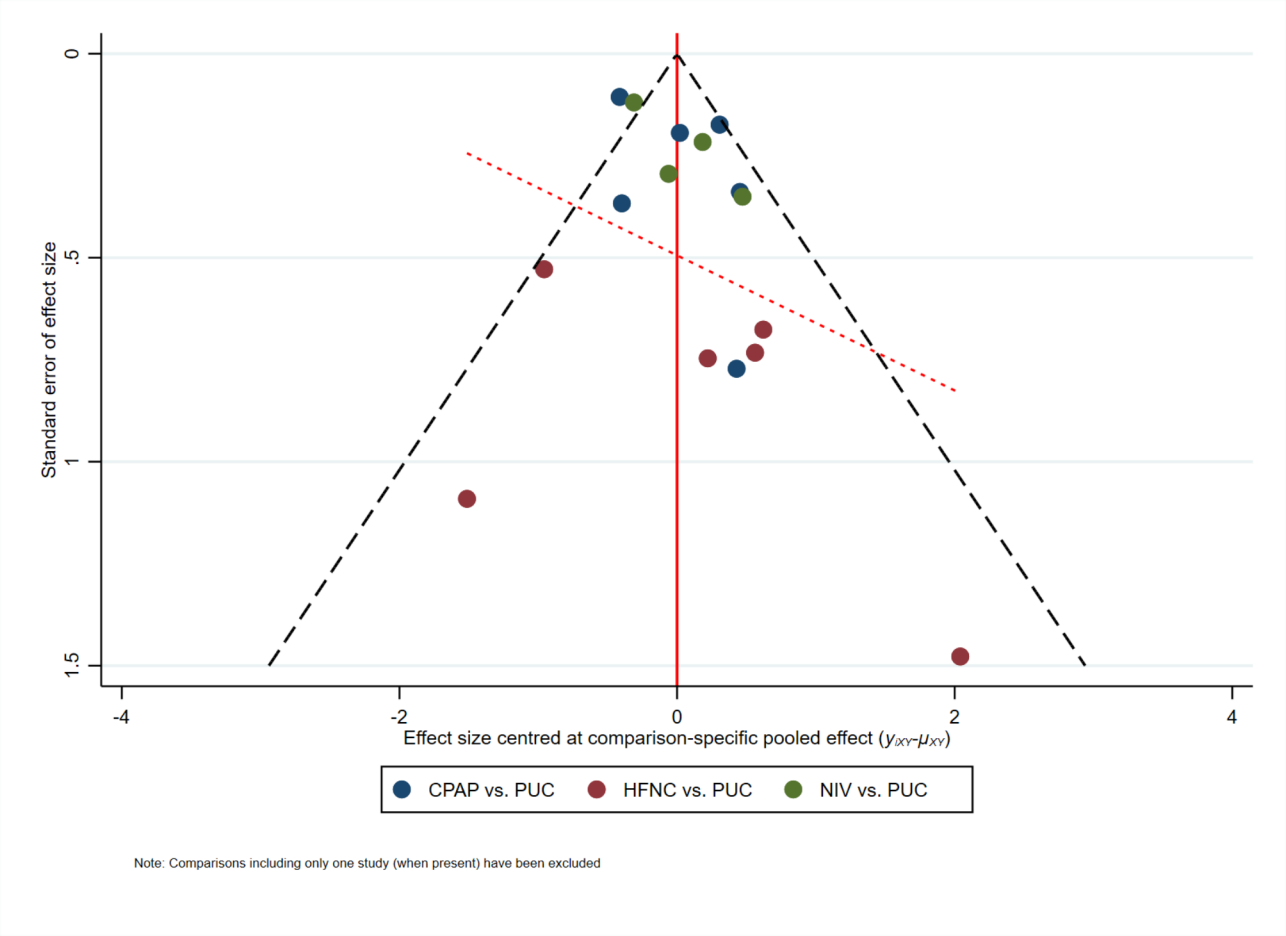
Assessment of small-study effects for the incidence of PPCs. PPCs postoperative pulmonary complications; CPAP continuous positive airway pressure; NIV noninvasive ventilation; HFNC high flow nasal cannula; PUC postoperative usual care

There were 14 trials [27–34, 36–41] reporting the incidence of PPCs, with the definition of PPCs largely varying across the included trials. We found no significant incoherence in the entire network (*P* = 0.638). The NMA results suggested that NIV compared with PUC significantly reduced the incidence of PPCs (low certainty) (Fig. 3; Table 1). As compared to PUC, the prophylactic use of HFNC (low certainty) or CPAP (very low certainty) was not associated with a significantly lower incidence of PPCs, despite exhibiting a downward trend (Fig. 3; Table 1). No significant differences among CPAP, NIV, and HFNC were observed for the primary outcome (Table S3, Additional File 2). According to the SUCRA estimates, NIV (83.0%) was the highest-ranked approach, followed by HFNC (62.5%), CPAP (44.3%), and PUC (10.2%). The best estimate of the treatment effect of NIV on the incidence of PPCs suggested an absolute reduction of 7.6% (95% CI: 1.6–11.8%) relative to PUC (Table 2). We found no evidence of small-study effects (Fig. 4). Given the limited included studies, we abandoned the scheduled plan of conducting a sensitivity analysis on the patient’s age.

**Table 1:** Treatment effect estimates and quality assessment for each direct comparison in all studied outcomes

Outcomes	I^2^ value (%)	Conventional pairwise meta-analysis	Direct estimate	Quality^1^	Indirect estimate	Quality^1^	NMA estimate	Quality^1^
**Incidence of PPCs** (Test for inconsistency in this entire network: *P* = 0.638)								
CPAP vs. PUC	73.1*	0.83 (0.53, 1.29)	0.85 (0.59, 1.23)	Low^2,3^	0.67 (0.13, 3.31)	Low^2,3^	0.85 (0.60, 1.20)	Very Low^2,3,5^
HFNC vs. PUC	41.5	0.85 (0.37, 1.95)	0.91 (0.47, 1.74)	Moderate^2^	0.59 (0.29, 1.20)	Very Low^2,3,4^	0.74 (0.46, 1.20)	Low^2,5^
NIV vs. PUC	59.9*	0.63 (0.44, 0.89)	0.63 (0.43, 0.90)	Low^2,3^	0.95 (0.44, 2.07)	Low^2,4^	0.67 (0.49, 0.93)	Low^2,3^
NIV vs. CPAP	NE	1.00 (0.23, 4.34)	1.00 (0.21, 4.86)	Moderate^2^	0.78 (0.48, 1.27)	Low^2,3^	0.80 (0.50, 1.26)	Low^2,5^
NIV vs. HFNC	NE	1.08 (0.83, 1.41)	1.08 (0.58, 2.05)	High	0.70 (0.33, 1.47)	Low^2,3^	0.91 (0.56, 1.46)	Low^5,6^
**Incidence of atelectasis** (Test for inconsistency in this entire network: *P* = 0.613)								
CPAP vs. PUC	44.4	0.97 (0.71, 1.34)	0.96 (0.67, 1.38)	Moderate^2^	0.63 (0.13, 3.11)	Low^2,3^	0.95 (0.67, 1.34)	Low^2,5^
HFNC vs. PUC	NE	0.49 (0.12, 2.05)	0.49 (0.11, 2.20)	Moderate^2^	NE	NE	0.49 (0.11, 2.20)	Low^2,5^
NIV vs. PUC	70.2*	0.59 (0.38, 0.94)	0.63 (0.43, 0.93)	Low^2,3^	0.96 (0.20, 4.70)	Moderate^2^	0.65 (0.45, 0.93)	Moderate^2^
NIV vs. CPAP	NE	1.00 (0.23, 4.34)	1.00 (0.21, 4.69)	Moderate^2^	0.66 (0.40, 1.09)	Low^2,3^	0.68 (0.43, 1.09)	Low^2,5^
**Reintubation** (Test for inconsistency in this entire network: *P* = 0.802)								
HFNC vs. PUC	53.8*	0.58 (0.12, 2.71)	0.60 (0.15, 2.44)	Low^2,3^	0.89 (0.06, 13.5)	Low^2,4^	0.74 (0.24, 2.27)	Very Low^2,3,5^
NIV vs. PUC	0	0.99 (0.72, 1.35)	0.87 (0.14, 5.31)	Moderate^2^	0.59 (0.05, 6.93)	Very Low^2,3,4^	0.82 (0.29, 2.34)	Low^2,5^
NIV vs. HFNC	NE	0.98 (0.70, 1.37)	0.98 (0.13, 7.43)	High	1.44 (0.15, 13.90)	Low^2.3^	1.11 (0.41, 3.01)	Low^5,6^
**Short-term mortality** (Test for inconsistency in this entire network: *P* = 0.623)								
CPAP vs. PUC	NE	3.55 (0.19, 66.89)	3.55 (0.19, 66.89)	Moderate^2^	NE	NE	3.55 (0.19, 66.89)	Very Low^2,7^
HFNC vs. PUC	0	0.61 (0.12, 3.11)	0.61 (0.12, 3.11)	Moderate^2^	1.23 (0.12, 12.45)	Low^2,4^	0.77 (0.20, 2.92)	Very Low^2,5,6^
NIV vs. PUC	0	1.00 (0.06, 15.64)	1.01 (0.11, 9.56)	Moderate^2^	0.50 (0.09, 2.77)	Low^2,4^	0.64 (0.16, 2.52)	Very Low^2,5,6^
NIV vs. HFNC	NE	0.81 (0.48, 1.39)	0.82 (0.48, 1.39)	Moderate^2^	1.66 (0.10, 26.82)	Moderate^2^	0.84 (0.50, 1.42)	Very Low^2,5,6^
**Length of ICU stay** (Test for inconsistency in this entire network: *P* = 0.980)								
CPAP vs. PUC	71.2*	-0.28 (-0.78, 0.22)	-0.57 (-1.17, 0.02)	Low^2,3^	-0.20 (-8.54, 8.13)	Moderate^2^	-0.57(-1.17, 0.02)	Low^2,5^
HFNC vs. PUC	9.3	-0.03 (-0.18, 0.13)	-0.09 (-0.64, 0.47)	Moderate^2^	0.04 (-1.23, 1.32)	Low^2,4^	-0.08 (-0.52, 0.37)	Very Low^2,5,6^
NIV vs. PUC	0	-0.02 (-0.20, 0.16)	0.05 (-0.74, 0.83)	Moderate^2^	-0.09 (-1.23, 1.05)	Low^2,4^	-0.01 (-0.56, 0.05)	Very Low^2,5,6^
NIV vs. CPAP	NE	0.20 (-8.09, 8.49)	0.12 (-8.12, 8.52)	Moderate^2^	0.57 (-0.24, 1.38)	Low^2,3^	0.57 (-0.23, 1.37)	Low^2,5^
NIV vs. HFNC	NE	0.0 (-0.6, 0.6)	0.0 (-1.01, 1.01)	High	0.13 (-0.83, 1.08)	Moderate^2^	0.07 (-0.53, 0.67)	Moderate^5^
**Length of in-hospital stay** (Test for inconsistency in this entire network: *P* = 0.484)								
CPAP vs. PUC	95.6*	-0.94 (-2.71, 0.83)	-0.8(-1.76, 0.16)	Low^2,3^	NE	NE	-0.8 (-1.76, 0.16)	Very Low^2,3,5^
HFNC vs. PUC	63.9*	-0.17 (-0.42, 0.09)	-0.38 (-1.09, 0.32)	Low^2,3^	-1.06 (-2.84, 0.71)	Very Low^2,3,4^	-0.47 (-1.11, 0.16)	Very Low^2,3,5^
NIV vs. PUC	80.5*	0.02 (-0.48, 0.43)	-0.06 (-0.67, 0.54)	Low^2,3^	0.62 (-1.20, 2.43)	Very Low^2,3,4^	0.0 (-0.56, 0.55)	Very Low^2,3,5,6^
NIV vs. HFNC	NE	1.00 (-0.27, 2.27)	1.0 (-0.67, 2.67)	High	0.32 (-0.61, 1.24)	Low^2,3^	0.47 (-0.32,1.26)	Moderate^5^

Categorical data are presented as relative risk and continuous data are presented as mean difference, with its corresponding 95% confidence intervals

No. number; NMA network meta-analysis; PPCs postoperative pulmonary complications; ICU intensive care unit; CPAP continuous positive airway pressure; NIV noninvasive ventilation; HFNC high flow nasal cannula; PUC postoperative usual care; NE not estimable

^*^ Indicating a significant heterogeneity among the included studies, with a *P* value less than 0.05 for the heterogeneity test;

^**1**^ Imprecision is only incorporated at the network level, not at the direct or indirect level

^**2**^ Rated down by one level for serious risk of bias

^**3**^ Rated down by one level for inconsistency because of substantial heterogeneity

^**4**^ Rated down by one level for intransitivity because the study by Stéphan et al. (reference no. 41) enrolled some patients with postoperative acute respiratory failure

^**5**^ Rated down by one level for imprecision because the wide 95% CI includes values favoring either treatment

^**6**^ Rated down by one level for incoherence between the direct and indirect estimates

^**7**^ Rated down by two levels for very serious imprecision because the very wide 95% CI includes values favoring either treatment

**Table 2:** Absolute treatment effect for the comparisons of various noninvasive respiratory support

Outcomes	No. of trials	Patients (n)	NMA estimate	RD per 1000 patients (95% CI)	NNT
**Incidence of PPCs** (Assumed control event rate across all trials in PUC arm was 23.1%)					
CPAP vs. PUC	5	620	0.85 (0.60, 1.20)	35 fewer (92 fewer to 46 more)	29
HFNC vs. PUC	6	788	0.74 (0.46, 1.20)	60 fewer (128 fewer to 46 more)	17
NIV vs. PUC	3	479	0.67 (0.49, 0.93)	76 fewer (118 fewer to 16 fewer)	13
NIV vs. CPAP	1	38	0.80 (0.50, 1.26)	46 fewer (115 fewer to 60 more)	22
NIV vs. HFNC	1	830	0.91 (0.56, 1.46)	21 fewer (102 fewer to 106 more)	48
**Incidence of atelectasis** (Assumed control event rate across all trials in PUC arm was 55.2%)					
CPAP vs. PUC	4	152	0.95 (0.67, 1.34)	28 fewer (182 fewer to 188 more)	36
HFNC vs. PUC	1	32	0.49 (0.11, 2.20)	282 fewer (491 fewer to 662 more)	4
NIV vs. PUC	3	479	0.65 (0.45, 0.93)	193 fewer (304 fewer to 39 fewer)	5
NIV vs. CPAP	1	38	0.68 (0.43, 1.09)	177 fewer (315 fewer to 50 more)	6
**Reintubation** (Assumed control event rate across all trials in PUC arm was 4.2%)					
HFNC vs. PUC	5	762	0.74 (0.24, 2.27)	11 fewer (32 fewer to 53 more)	91
NIV vs. PUC	2	379	0.82 (0.29, 2.34)	8 fewer (30 fewer to 56 more)	125
NIV vs. HFNC	1	830	1.11 (0.41, 3.01)	5 more (25 fewer to 84 more)	200
**Short-term mortality** (Assumed control event rate across all trials in PUC arm was 1.0%)					
CPAP vs. PUC	1	108	3.55 (0.19, 66.89)	26 more (8 fewer to 686 more)	38
HFNC vs. PUC	3	534	0.77 (0.20, 2.92)	2 fewer (8 fewer to 19 more)	500
NIV vs. PUC	2	379	0.64 (0.16, 2.52)	4 fewer (8 fewer to 15 more)	250
NIV vs. HFNC	1	830	0.84 (0.50, 1.42)	2 fewer (5 fewer to 4 more)	500

No. number; NMA network meta-analysis; RD risk difference; CI confidence interval; NNT number needed to treat; PPCs postoperative pulmonary complications; CPAP continuous positive airway pressure; NIV noninvasive ventilation; HFNC high flow nasal cannula; PUC postoperative usual care

### Secondary outcomes

Seven trials [27–29, 37–40] reported data on the incidence of atelectasis. No significant incoherence existed in the network. Compared with PUC, NIV had a beneficial effect on the incidence of atelectasis (moderate certainty) (Fig. 3), and the absolute risk reduction was 19.3% (95% CI: 3.9–30.4%) (Table 2). No positive results were observed in other direct or indirect comparisons for the incidence of atelectasis (Table S3) (Additional File 2). In addition, as shown in Table 2 and Table S3, no statistical difference was observed among the four treatments in terms of reintubation, short-term mortality, the length of ICU stay, or the length of in-hospital stay. The associated network plots and forest plots were presented in Figures S1-S4 (Additional File 2).

## Discussion

This systematic NMA evaluated the relative effectiveness of prophylactic noninvasive respiratory strategies in the immediate postoperative period after cardiac surgery. The principal findings suggested the superiority of NIV over PUC in reducing the incidence of PPCs and the incidence of atelectasis. However, prophylactic CPAP or HFNC compared with PUC had no significant beneficial effect on the incidence of PPCs or atelectasis. No one particular noninvasive respiratory approach is superior to another in terms of primary or secondary outcomes. The SUCRA statistics indicated that prophylactic NIV compared with PUC is probably the most effective approach to prevent PPCs in patients undergoing cardiac surgery.

Postoperative noninvasive respiratory support has always been an important clinical scenario that surgeons, anesthesiologists, and intensivists are concerned about. The most recent release of European Respiratory Society guidelines recommended the application of NIV for treating postoperative patients with acute respiratory failure [42]. However, there is no clear recommendation on the prophylactic use of NPPV in the immediate postoperative period. In this current NMA, we found a beneficial effect of postoperative NIV on the incidence of PPCs (primarily atelectasis), which was inconsistent with the results of the latest meta-analysis which did not support the routine use of postoperative NPPV to prevent PPCs [43]. Distinct target populations might contribute to these conflicting findings. The studied subjects in the previous study [43] were patients undergoing major surgery, primarily abdominal surgery, and in our study were patients undergoing cardiac surgery. Theoretically, patients undergoing cardiac surgery are at higher risk of developing PPCs than those undergoing abdominal surgery because the former commonly suffer from various cardiovascular and pulmonary dysfunction (such as pulmonary oedema) at the preoperative stage. In this case, prophylactic NIV exhibits the ability to reduce left ventricular pre- and afterload and improve cardiac performance by the elevation of intrathoracic pressure [7, 44], finally resulting in a reduced incidence of PPCs.

However, this NMA did not identify any effect of NPPV (including NIV and CPAP) on the reintubation or mortality, which was not dissimilar to the previous study [43]. The negative findings are not unexpected because both reintubation rate and short-term mortality are quite low so the small sample size in the included trials might have no sufficient statistical power to detect a slight reduction in both outcomes. More importantly, potential self-inflicted lung injury and delayed reintubation might counterbalance the survival benefit resulting from reduced PPCs [45, 46]. These potential harms are common to all noninvasive oxygenation strategies. In postoperative patients with spontaneous breathing, noninvasive respiratory support typically leads to higher-than-targeted tidal volumes, resulting in high transpulmonary pressures and self-inflicted lung injury [47, 48]. Furthermore, oxygenation improvement with the use of noninvasive respiratory support might disguise signs of respiratory distress for an extended period in those patients who would finally fail on noninvasive respiratory treatment, ultimately leading to delayed reintubation and increased mortality [49].

As a promising alternative to NPPV, post-extubation HFNC has been recommended as an effective noninvasive respiratory strategy in various clinical situations, including the postoperative period. Based on evidence from a recent meta-analysis suggesting the benefits of postoperative HFNC in reducing reintubation and escalation of respiratory support [13], the latest practice guideline recommended the use of HFNC compared to PUC to prevent respiratory failure in the immediate postoperative period in high-risk and/or obese patients undergoing cardiothoracic surgery [50]. However, the current NMA found a downward trend of PPCs events with the preventive use of HFNC compared with PUC, without a statistical significance. By adding a recently published trial [33], our study demonstrated a neutral effect of HFNC on reintubation or mortality as compared to PUC. Our results were consistent with the findings of the latest meta-analysis [43]. These findings are not surprising because the low level of positive airway pressure generated by HFNC, relative to NPPV, might be insufficient to produce a significant impact on alveolar collapse and transpulmonary pressures in postoperative patients, and consequently exhibits no significant effect on cardiorespiratory performance. However, the very low to low certainty of evidence on the efficacy of HFNC should be interpreted with caution. At present, a conducting, well-designed, multi-center, international RCT is anticipated to draw a more reliable conclusion on the efficacy of postoperative HFNC [51].

The current NMA has several strengths. To the best of our knowledge, this study is the first NMA on the efficacy of noninvasive respiratory support applied in the postoperative period. This NMA manifests an intrinsic advantage of allowing for the comparison of multiple treatments simultaneously and improving the precision by combining direct and indirect evidence. Moreover, this study adds the latest published data and provides a greater number of included trials and subjects (16 trials with 3011 patients) than the previous meta-analyses [11–13]. Of note, there are at least 2 trials [29, 39] that were missed in a previous study [11], and 1 trial [34] was missed in another study [13]. The incomplete data synthesis would downgrade the credibility of their evidence to some extent. In addition, the calculation of the absolute treatment effect facilitates a better understanding of the clinical significance of noninvasive respiratory support.

However, several limitations in this study should be recognized. First, the varied definitions of PPCs represent the main cause of heterogeneities among the included trials. Although the varied PPCs definitions might cause an underestimation or overestimation of the true incidence of PPCs, it should have equally affected the different groups analyzed. Furthermore, we specifically evaluated the individual outcome of atelectasis because PPCs is a composite outcome that cannot precisely describe the characteristic of individual complications. Second, we did not perform subgroup analyses to clarify some potential effects modifiers, including the interfaces used, smoking, obesity, age, surgical approach, and the risk of developing PPCs, due to the limited information, which must be taken into consideration when interpreting the findings. Third, the lack of blinding could pose a potential bias in subjective outcomes (e.g., the incidence of PPCs) in the majority of the included trials. We downgraded the certainty of the evidence for these subjective outcomes in the domain of risk of bias, even though it is unrealistic to blind the treating clinicians to treatment allocation among noninvasive respiratory interventions. Finally, the findings of our study are only applicable to the prophylactic use in the immediate postoperative period after cardiac surgery, not to other clinical scenarios. In addition, no report on the safety, adverse outcomes, or cost may affect clinical practice or policy decisions.

## Conclusion

This systematic NMA supports the routine use of prophylactic NIV in the immediate postoperative period to prevent PPCs (primarily atelectasis) in patients undergoing cardiac surgery. The current NMA evidence presents important clinical implications for surgeons, anesthesiologists, and intensivists, and should prompt a re-evaluation of practice guidelines with respect to the postoperative use of noninvasive respiratory support. Given the overall low certainty of the evidence, further high-quality research is warranted to better understand the relative benefits of each noninvasive ventilatory support.

## Electronic supplementary material

Below is the link to the electronic supplementary material.

Supplementary Material 1

Supplementary Material 2

## Data Availability

All data generated or analysed during this study are included in this published article (and its supplementary information files).

## Abbreviations

PPCs: Postoperative pulmonary complications
NPPV: Noninvasive positive pressure ventilation
NIV: Noninvasive ventilation
CPAP: Continuous positive airway pressure
HFNC: High flow nasal cannula
PUC: Postoperative usual care
NMA: Network meta-analysis
PRISMA: The preferred reporting items for systematic reviews and meta-analyses
CENTRAL: Cochrane controlled clinical trial register
RCTs: Randomized controlled trials
ICU: Intensive care unit
RR: Relative risk
MD: Mean differences
CI: Confidence intervals
SUCRA: The surface under the cumulative ranking curve
