# Supplementary material for: Prophylactic noninvasive respiratory support in the immediate postoperative period after cardiac surgery - a systematic review and network meta-analysis
Source: BMC Pulm Med. 2023 Jun 28;23:233. doi: 10.1186/s12890-023-02525-1 (PMC10303297; doi:10.1186/s12890-023-02525-1)
Supplement: Supplementary file 1 — Supplementary Material 1 [file 12890_2023_2525_MOESM1_ESM.docx]

# Detailed search strategies for each database

**PUBMED (271 RECORDS)**

1. (((((((((Noninvasive ventilation[MeSH Terms]) OR (Noninvasive Ventilation*)) OR (Ventilation*, Noninvasive)) OR (Non-Invasive Ventilation*)) OR (Ventilation*, Non-Invasive)) OR (Non Invasive Ventilation*)) OR (Ventilation*, Non Invasive)) OR (noninvasive positive pressure ventilation)) OR (non-invasive positive pressure ventilation)) OR (NIPPV) (14761 records)
2. (((((((((((((Continuous Positive Airway Pressure[MeSH Terms]) OR (CPAP)) OR (CPAP Ventilation)) OR (Ventilation, CPAP)) OR (Nasal Continuous Positive Airway Pressure)) OR (nCPAP Ventilation)) OR (Ventilation, nCPAP)) OR (Biphasic Continuous Positive Airway Pressure)) OR (BiPAP Biphasic Positive Airway Pressure)) OR (BiPAP Bilevel Positive Airway Pressure)) OR (Biphasic Positive Airway Pressure)) OR (Bilevel Continuous Positive Airway Pressure)) OR (Bilevel Positive Airway Pressure)) OR (BiPAP) (17270 records)
3. ((((((Cannula[MeSH Terms]) OR (Nasal Cannula)) OR (Cannula, Nasal)) OR (Nasal Cannulae)) OR (Cannulae, Nasal)) AND ((high-flow) OR (high flow))) OR (((HFNC) OR (HHFNC)) OR (HHFN)) (2377 records)
4. ((high-flow) OR (high flow)) AND (((((((Oxygen inhalation therapy[MeSH Terms]) OR (Inhalation Therapy, Oxygen)) OR (Inhalation Therapies, Oxygen)) OR (Oxygen Inhalation Therapies)) OR (Therapies, Oxygen Inhalation)) OR (Therapy, Oxygen Inhalation)) OR (Oxygen therapy)) (4144 records)
5. #3 OR #4 (5268 records)
6. #1 OR #2 OR #5 (33563 reocrds)
7. (((Postoperative Period[MeSH Terms]) OR (Period, Postoperative)) OR (Periods, Postoperative)) OR (Postoperative Periods) (194114 reocrds)
8. ((((((((((((Thoracic Surgery[MeSH Terms]) OR (Cardiac Surgical Procedures[MeSH Terms])) OR (Surgery, Thoracic)) OR (Surgery, Cardiac)) OR (Surgery, Heart)) OR (Heart Surgery)) OR (Cardiac Surgery)) OR (Procedure*, Cardiac Surgical)) OR (Surgical Procedure*, Cardiac)) OR (Surgical Procedure*, Heart)) OR (Cardiac Surgical Procedure*)) OR (Heart Surgical Procedure*)) OR (Procedure*, Heart Surgical) (748436 records)
9. #6 AND #7 AND #8 (271 records)

**EMBASE (76 RECORDS)**

1. noninvasive AND ventilation OR (noninvasive AND ventilation*) OR (ventilation*, AND noninvasive) OR ('non invasive' AND ventilation*) OR (ventilation*, AND 'non invasive') OR (non AND invasive AND ventilation*) OR (ventilation*, AND non AND invasive) OR (noninvasive AND positive AND pressure AND ventilation) OR ('non invasive' AND positive AND pressure AND ventilation) OR nippv (32010 records)
2. continuous AND positive AND airway AND pressure OR cpap OR (cpap AND ventilation) OR (ventilation, AND cpap) OR (nasal AND continuous AND positive AND airway AND pressure) OR (ncpap AND ventilation) OR (ventilation, AND ncpap) OR (biphasic AND continuous AND positive AND airway AND pressure) OR (bipap AND biphasic AND positive AND airway AND pressure) OR (bipap AND bilevel AND positive AND airway AND pressure) OR (biphasic AND positive AND airway AND pressure) OR (bilevel AND continuous AND positive AND airway AND pressure) OR (bilevel AND positive AND airway AND pressure) OR bipap (30140 records)
3. nasal AND cannula OR (cannula, AND nasal) OR (nasal AND cannulae) OR (cannulae, AND nasal) (10105 records)
4. 'high flow' OR (high AND flow) (326616 records)
5. #3 AND #4 (5138 records)
6. oxygen AND inhalation AND therapy OR (inhalation AND therapies, AND oxygen) OR (inhalation AND therapy, AND oxygen) OR (oxygen AND inhalation AND therapies) OR (therapies, AND oxygen AND inhalation) OR (therapy, AND oxygen AND inhalation) OR (oxygen AND therapy) (252952 records)
7. #4 AND #6 (9547 records)
8. #5 OR #7 (11499 records)
9. #1 OR #2 OR #8 (65853 records)
10. postoperative AND period OR (period, AND postoperative) OR (periods, AND postoperative) OR (postoperative AND periods) (411883 records)
11. thoracic AND surgery OR (surgery, AND thoracic) OR (surgery, AND cardiac) OR (surgery, AND heart) OR (heart AND surgery) OR (cardiac AND surgery) OR (procedure*, AND cardiac AND surgical) OR (surgical AND procedure*, AND cardiac) OR (surgical AND procedure*, AND heart) OR (cardiac AND surgical AND procedure*) OR (heart AND surgical AND procedure*) OR (procedure*, AND heart AND surgical) (942717 records)
12. #9 AND #10 AND #11 AND 'randomized controlled trial'/de (76 records)

**Web of Science (122 RECORDS)**

1. Noninvasive ventilation (All Fields) or Noninvasive Ventilation* (All Fields) or Ventilation*, Noninvasive (All Fields) or Non-Invasive Ventilation* (All Fields) or Ventilation*, Non-Invasive (All Fields) or Non Invasive Ventilation* (All Fields) or Ventilation*, Non Invasive (All Fields) or noninvasive positive pressure ventilation (All Fields) or non-invasive positive pressure ventilation (All Fields) or nippv (All Fields) (15,867 results)
2. Continuous Positive Airway Pressure (All Fields) or CPAP (All Fields) or CPAP Ventilation (All Fields) or Ventilation, CPAP (All Fields) or Nasal Continuous Positive Airway Pressure (All Fields) or nCPAP Ventilation (All Fields) or Ventilation, nCPAP (All Fields) or Biphasic Continuous Positive Airway Pressure (All Fields) or BiPAP Biphasic Positive Airway Pressure (All Fields) or BiPAP Bilevel Positive Airway Pressure (All Fields) or Biphasic Positive Airway Pressure (All Fields) or Bilevel Continuous Positive Airway Pressure (All Fields) or Bilevel Positive Airway Pressure (All Fields) or BiPAP (All Fields) (17947 records)
3. Nasal Cannula (All Fields) or Cannula, Nasal (All Fields) or Nasal Cannulae (All Fields) or Cannulae, Nasal (All Fields) (3599 records)
4. high-flow (All Fields) or high flow (All Fields) (829976 records)
5. #3 AND #4 (2424 records)
6. oxygen inhalation therapy (All Fields) or Inhalation Therapy, Oxygen (All Fields) or Inhalation Therapies, Oxygen (All Fields) or Oxygen Inhalation Therapies (All Fields) or Therapies, Oxygen Inhalation (All Fields) or Therapy, Oxygen Inhalation (All Fields) or oxygen therapy (All Fields) (71697 records)
7. #4 AND #6 (4030 records)
8. #5 OR #7 (5199 records)
9. #1 OR #2 OR #8 (34,855 records)
10. Postoperative Period (All Fields) or Period, Postoperative (All Fields) or Periods, Postoperative (All Fields) or Postoperative Periods (All Fields) (77336 records)
11. Thoracic Surgery (All Fields) or Surgery, Thoracic (All Fields) or Surgery, Cardiac (All Fields) or Surgery, Heart (All Fields) or Heart Surgery (All Fields) or Cardiac Surgery (All Fields) or Cardiac Surgical Procedures (All Fields) or Procedure*, Cardiac Surgical (All Fields) or Surgical Procedure*, Cardiac (All Fields) or Surgical Procedure*, Heart (All Fields) or Cardiac Surgical Procedure* (All Fields) or Heart Surgical Procedure* (All Fields) or Procedure*, Heart Surgical (All Fields) (489227 records)
12. #9 AND #10 AND #11 and Articles (Document Types) (122 records)

**Cochrane Controlled Clinical Trial Register (CENTRAL) (136 RECORDS)**

1. [Continuous Positive Airway Pressure] explode all trees (1222 records)
2. (CPAP):ti,ab,kw OR (CPAP Ventilation):ti,ab,kw OR (Ventilation, CPAP):ti,ab,kw OR (Nasal Continuous Positive Airway Pressure):ti,ab,kw OR (nCPAP Ventilation):ti,ab,kw (5553 records)
3. (Biphasic Continuous Positive Airway Pressure):ti,ab,kw OR (BiPAP Biphasic Positive Airway Pressure):ti,ab,kw OR (Bilevel Continuous Positive Airway Pressure):ti,ab,kw OR (BiPAP Bilevel Positive Airway Pressure):ti,ab,kw OR (Biphasic Positive Airway Pressure):ti,ab,kw (316 records)
4. #1 OR #2 OR #3 (5851 records)
5. [Cannula] explode all trees (147 records)
6. (Nasal Cannula) OR (Cannula*, Nasal) OR (Nasal Cannula*) (1951 records)
7. #5 OR #6 (1971 records)
8. (high-flow) OR (high flow) (18418 records)
9. #7 AND #8 (1275 records)
10. (HFNC) OR (HHFNC) OR (HHFN) (614 records)
11. [Oxygen Inhalation Therapy] explode all trees (1681 records)
12. (Inhalation Therapy, Oxygen) OR (Inhalation Therapies, Oxygen) OR (Oxygen Inhalation Therapies) OR (Therapies, Oxygen Inhalation) OR ((Therapy, Oxygen Inhalation) OR (oxygen therapy)) (21954 records)
13. #11 OR #12 (22032 records)
14. #8 AND #13 (2746 records)
15. #9 OR #10 OR #14 (3317 records)
16. [Noninvasive Ventilation] explode all trees (317 records)
17. (Noninvasive Ventilation*):ti,ab,kw OR (Ventilation*, Noninvasive):ti,ab,kw OR (Non-Invasive Ventilation*):ti,ab,kw OR (non*invasive positive pressure ventilation):ti,ab,kw OR (NIPPV):ti,ab,kw (4411 records)
18. #16 OR #17 (4411 records)
19. #4 OR #15 OR #18 (11735 records)
20. [Postoperative Period] explode all trees (6196 records)
21. (Postoperative Period):ti,ab,kw OR (Periods, Postoperative):ti,ab,kw OR (Period, Postoperative):ti,ab,kw OR (Postoperative Periods):ti,ab,kw (36238 records)
22. #20 OR #21(37220 records)
23. [Thoracic Surgery] explode all trees (174 records)
24. [Cardiac Surgical Procedures] explode all trees (13320 records)
25. (Thoracic Surgery):ti,ab,kw OR (Surgery, Thoracic):ti,ab,kw OR (Surgery, Cardiac):ti,ab,kw OR (Surgery, Heart):ti,ab,kw AND (Cardiac Surgery):ti,ab,kw (25056 records)
26. (Procedure*, Cardiac Surgical):ti,ab,kw OR (Surgical Procedure*, Cardiac):ti,ab,kw OR (Surgical Procedure*, Heart):ti,ab,kw OR (Cardiac Surgical Procedure*):ti,ab,kw AND (Heart Surgical Procedure*):ti,ab,kw (2977 records)
27. #23 OR #24 OR #25 OR #26 (33319 records)
28. #19 AND #22 AND #27 (136 records)

# Reasons for excluding ineligible studies

| **Reasons for exclusion** | **Studies** |
| --- | --- |
| Studies that did not report interested outcomes (6 studies) | Gust/1996 [1];  Lopes/2008 [2];  Mazullo Filho/2010 [3];  Roceto Ldos/2014 [4];  Sah/2017 [5];  Nery/2012 [6] |
| Studies that enrolled patients with postoperative respiratory failure (5 studies) | Coimbra/2007 [7];  Zhu/2013 [8];  Yang/2016 [9];  Laverdure/2019 [10];  Theologou/2021 [11] |
| Studies without randomized controlled design (3 studies) | Boeken/2010 [12];  Marcondi/2018 [13];  Liu/2020 [14]; |
| Noninvasive respiratory support had also been used before surgery (2 studies) | Perrin/2007 [15];  Guerra/2018 [16]; |
| Noninvasive respiratory support was not used in the immediate postoperative period after surgery (3 studies) | Pantoni/2016 [17];  Windmöller/2020 [18];  Silva/2022 [19]; |
| Noninvasive respiratory support was not used for prophylactic purpose | Pasquina/2004 [20]; |
| Irrelevant studies (14 studies):  10 studies enrolled patients undergone lung resection surgery;  2 studies enrolled patients undergone open repair of thoracoabdominal aortic aneurysms;  1 study enrolled patients undergone thoracoabdominal surgery;  1 study inappropriately excluded those patients who received reintubation and non-invasive mechanical ventilation | Puente-Maestú/2021 [21]; Barbagallo/2012 [22]; Garutti/2014 [23]; Palleschi/2018 [24]; Ansari/2016 [25]; Brainard/2017 [26]; Pennisi/2019 [27]; Yu/2017 [28]; Liao/2010 [29]; Lorut/2014 [30];  Kindgen-Milles/2005 [31];  Mamo/2019 [32]  Fagevik Olsén/2002 [33];  de Araújo-Filho/2017 [34]; |
| Conference abstract (1 study) | De Novaes/2018 [35] |

**Reference**

1. Gust R, Gottschalk A, Schmidt H, Böttiger BW, Böhrer H, Martin E. Effects of continuous (CPAP) and bi-level positive airway pressure (BiPAP) on extravascular lung water after extubation of the trachea in patients following coronary artery bypass grafting. Intensive Care Med. 1996;22(12):1345-50.
2. Lopes CR, Brandão CM, Nozawa E, Auler JO Jr. Benefits of non-invasive ventilation after extubation in the postoperative period of heart surgery. Rev Bras Cir Cardiovasc. 2008;23(3):344-50.
3. Mazullo Filho JB, Bonfim VJ, Aquim EE. Noninvasive mechanical ventilation in immediate postoperative cardiac surgery patients. Rev Bras Ter Intensiva. 2010;22(4):363-8.
4. Roceto Ldos S, Galhardo FD, Saad IA, Toro IF. Continuous positive airway pressure (CPAP) after lung resection: a randomized clinical trial. Sao Paulo Med J. 2014;132(1):41-7.
5. Sah HK, Akcil EF, Tunali Y, Vehid H, Dilmen OK. Efficacy of continuous positive airway pressure and incentive spirometry on respiratory functions during the postoperative period following supratentorial craniotomy: A prospective randomized controlled study. J Clin Anesth. 2017;42:31-35.
6. Nery FP, Lopes AJ, Domingos DN, Cunha RF, Peixoto MG, Higa C, et al. CPAP increases 6-minute walk distance after lung resection surgery. Respir Care. 2012;57(3):363-9.
7. Coimbra VR, Lara Rde A, Flores EG, Nozawa E, Auler JO Jr, Feltrim MI. Application of noninvasive ventilation in acute respiratory failure after cardiovascular surgery. Arq Bras Cardiol. 2007;89(5):270-6, 298-305.
8. Zhu GF, Wang DJ, Liu S, Jia M, Jia SJ. Efficacy and safety of noninvasive positive pressure ventilation in the treatment of acute respiratory failure after cardiac surgery. Chin Med J (Engl). 2013;126(23):4463-9.
9. Yang Y, Liu N, Sun L, Zhou Y, Yang Y, Shang W, et al. Noninvasive Positive-Pressure Ventilation in Treatment of Hypoxemia After Extubation Following Type-A Aortic Dissection. J Cardiothorac Vasc Anesth. 2016;30(6):1539-1544.
10. Laverdure F, Genty T, Rezaiguia-Delclaux S, Herve P, Stephan F. Ultrasound Assessment of Respiratory Workload With High-Flow Nasal Oxygen Versus Other Noninvasive Methods After Chest Surgery. J Cardiothorac Vasc Anesth. 2019;33(11):3042-3047.
11. Theologou S, Ischaki E, Zakynthinos SG, Charitos C, Michopanou N, Patsatzis S, et al. High Flow Oxygen Therapy at Two Initial Flow Settings versus Conventional Oxygen Therapy in Cardiac Surgery Patients with Postextubation Hypoxemia: A Single-Center, Unblinded, Randomized, Controlled Trial. J Clin Med. 2021;10(10):2079.
12. Boeken U, Schurr P, Kurt M, Feindt P, Lichtenberg A. Early reintubation after cardiac operations: impact of nasal continuous positive airway pressure (nCPAP) and noninvasive positive pressure ventilation (NPPV). Thorac Cardiovasc Surg. 2010;58(7):398-402.
13. Marcondi NO, Rocco IS, Bolzan DW, Pauletti HO, Begot I, Anjos NR, et al. Noninvasive Ventilation After Coronary Artery Bypass Grafting in Subjects With Left-Ventricular Dysfunction. Respir Care. 2018;63(7):879-885.
14. Liu K, Hao GW, Zheng JL, Luo JC, Su Y, Hou JY, et al. Effect of Sequential Noninvasive Ventilation on Early Extubation After Acute Type A Aortic Dissection. Respir Care. 2020;65(8):1160-1167.
15. Perrin C, Jullien V, Vénissac N, Berthier F, Padovani B, Guillot F, et al. Prophylactic use of noninvasive ventilation in patients undergoing lung resectional surgery. Respir Med. 2007;101(7):1572-8.
16. Guerra Hernández E, Rodríguez Pérez A, Freixinet Gilard J, Martín Álamo MN, Escudero Socorro M, Rodríguez Suárez P, et al. Prophylactic use of non-invasive mechanical ventilation in lung resection. Eur Rev Med Pharmacol Sci. 2018;22(1):190-198.
17. Pantoni CB, Di Thommazo-Luporini L, Mendes RG, Caruso FC, Mezzalira D, Arena R, et al. Continuous Positive Airway Pressure During Exercise Improves Walking Time in Patients Undergoing Inpatient Cardiac Rehabilitation After Coronary Artery Bypass Graft Surgery: A RANDOMIZED CONTROLLED TRIAL. J Cardiopulm Rehabil Prev. 2016;36(1):20-7.
18. Windmöller P, Bodnar ET, Casagrande J, Dallazen F, Schneider J, Berwanger SA, et al. Physical Exercise Combined With CPAP in Subjects Who Underwent Surgical Myocardial Revascularization: A Randomized Clinical Trial. Respir Care. 2020;65(2):150-157.
19. Silva AMVD, de Nardi AT, de Almeida Righi G, Nascimento JR, Lima RM, Signori LU. Bilevel positive airway pressure improves the autonomic balance in the postoperative period following cardiac surgery: a randomized trial. Fisioterapia e pesquisa. 2022; 29(1): 4-10.
20. Pasquina P, Merlani P, Granier JM, Ricou B. Continuous positive airway pressure versus noninvasive pressure support ventilation to treat atelectasis after cardiac surgery. Anesth Analg. 2004;99(4):1001-1008.
21. Puente-Maestú L, López E, Sayas J, Alday E, Planas A, Parise DJ; PI12/02734 study group. The effect of immediate postoperative Boussignac CPAP on adverse pulmonary events after thoracic surgery: A multicentre, randomised controlled trial. Eur J Anaesthesiol. 2021;38(2):164-170.
22. Barbagallo M, Ortu A, Spadini E, Salvadori A, Ampollini L, Internullo E, et al. Prophylactic use of helmet CPAP after pulmonary lobectomy: a prospective randomized controlled study. Respir Care. 2012;57(9):1418-24.
23. Garutti I, Puente-Maestu L, Laso J, Sevilla R, Ferrando A, Frias I, et al. Comparison of gas exchange after lung resection with a Boussignac CPAP or Venturi mask. Br J Anaesth. 2014;112(5):929-35.
24. Palleschi A, Privitera E, Lazzeri M, Mariani S, Rosso L, Tosi D, et al. Prophylactic continuous positive airway pressure after pulmonary lobectomy: a randomized controlled trial. J Thorac Dis. 2018;10(5):2829-2836.
25. Ansari BM, Hogan MP, Collier TJ, Baddeley RA, Scarci M, Coonar AS, et al. A randomized controlled trial of high-flow nasal oxygen (Optiflow) as part of an enhanced recovery program after lung resection surgery. Ann Thorac Surg. 2016;101(2):459-64.
26. Brainard J, Scott BK, Sullivan BL, Fernandez-Bustamante A, Piccoli JR, Gebbink MG, et al. Heated humidified high-flow nasal cannula oxygen after thoracic surgery - A randomized prospective clinical pilot trial. J Crit Care. 2017;40:225-228.
27. Pennisi MA, Bello G, Congedo MT, Montini L, Nachira D, Ferretti GM, et al. Early nasal high-flow versus Venturi mask oxygen therapy after lung resection: a randomized trial. Crit Care. 2019;23(1):68.
28. Yu Y, Qian X, Liu C, Zhu C. Effect of high-flow nasal cannula versus conventional oxygen therapy for patients with thoracoscopic lobectomy after extubation. Can Respir J. 2017; 2017:7894631.
29. Liao G, Chen R, He J. Prophylactic use of noninvasive positive pressure ventilation in post-thoracic surgery patients: A prospective randomized control study. J Thorac Dis. 2010;2(4):205-9.
30. Lorut C, Lefebvre A, Planquette B, Quinquis L, Clavier H, Santelmo N, et al. Early postoperative prophylactic noninvasive ventilation after major lung resection in COPD patients: a randomized controlled trial. Intensive Care Med. 2014;40(2):220-227.
31. Kindgen-Milles D, Müller E, Buhl R, Böhner H, Ritter D, Sandmann W, et al. Nasal-continuous positive airway pressure reduces pulmonary morbidity and length of hospital stay following thoracoabdominal aortic surgery. Chest. 2005;128(2):821-8.
32. Mamo D, Zangrillo A, Cabrini L, Leggieri C, Olper L, Monaco F, et al. Noninvasive ventilation after thoracoabdominal aortic surgery: a pilot randomized controlled trial. J Cardiothorac Vasc Anesth. 2019;33(6):1639-1645.
33. Fagevik Olsén M, Wennberg E, Johnsson E, Josefson K, Lönroth H, Lundell L. Randomized clinical study of the prevention of pulmonary complications after thoracoabdominal resection by two different breathing techniques. Br J Surg. 2002;89(10):1228-34.
34. de Araújo-Filho AA, de Cerqueira-Neto ML, de Assis Pereira Cacau L, Oliveira GU, Cerqueira TCF, de Santana-Filho VJ. Effect of prophylactic non-invasive mechanical ventilation on functional capacity after heart valve replacement: a clinical trial. Clinics (Sao Paulo). 2017;72(10):618-623.
35. De Novaes A, De Andrade AD, Campos S, Modolo N, Goncalves C, Morais C, et al. Electrical impedance tomography in evaluating the effects of noninvasive ventilation in the postoperative cardiac surgery: randomized controlled clinical trial. Eur Respir J. 2018, 52.
