# Supplementary material for: Prophylactic noninvasive respiratory support in the immediate postoperative period after cardiac surgery - a systematic review and network meta-analysis
Source: BMC Pulm Med. 2023 Jun 28;23:233. doi: 10.1186/s12890-023-02525-1 (PMC10303297; doi:10.1186/s12890-023-02525-1)
Supplement: Supplementary file 2 — Supplementary Material 2 [file 12890_2023_2525_MOESM2_ESM.docx]

**Prophylactic noninvasive respiratory support in the immediate postoperative period after cardiac surgery - a systematic review and network meta-analysis**

**Table of contents**

[Table S1. Characteristics of each included study. 2](#_Toc134940674)

[Table S2. Outcome measures in each included study. 9](#_Toc134940675)

[Table S3. League table for networks of various noninvasive respiratory support. 12](#_Toc134940676)

[Figure S1. Forest plot and network plot for the reintubation 14](#_Toc134940677)

[Figure S2. Forest plot and network plot for the short-term mortality 15](#_Toc134940678)

[Figure S3. Forest plot and network plot for the length of ICU stay 16](#_Toc134940679)

[Figure S4. Forest plot and network plot for the length of in-hospital stay 17](#_Toc134940680)

## Table S1. Characteristics of each included study.

| First author /Year | Design (location) | Sample size | | Type of surgery and urgency | Experimental group (EG) | Control group (CG) | Mean age  (year) | Mean BMI  (kg/m^2^) | Duration of surgery or ECC (min or h) | | Current somker, n (%) | Definition of PPCs |
| --- | --- | --- | --- | --- | --- | --- | --- | --- | --- | --- | --- | --- |
|  |  |  |  |  |  |  |  |  | EG | CG |  |  |
| Al-Mutairi/2012 | Single-center (Saudi Arabia) | 108 | Cardiac surgery; Elective | | **CPAP** (supporting pressure: 4-6 cmH_2_O) via mask was initiated after extubation for 0.5 h every 2 h or 4 h for 3 days | **Postoperative usual care** (including incentive spirometry, COT) | 62 | NR | NR | NR | NR | No clear definition of PPCs, and no record of any composites of PPCs that defined by this NMA. |
| Jousela/1994 | Single-center (USA) | 30 | Cardiac surgery (CABG); Elective | | **CPAP** (supporting pressure: 7.4 mmHg) with mask was used for 8 h following extubation | **Postoperative usual care** (including pulmonary therapy, COT) | 53 | 26.7 | 304 | 286 | 8 (27) | This study had no clear definition of PPCs, but it presented data on the incidence of atelectasis. |
| Pinilla/1990 | Single-center (Canada) | 58 | Cardiac surgery (CABG using CPB); Elective | | **CPAP** (supporting pressure: 5 or 7.5 cmH_2_O) via a nose or face mask was applied immediately after extubation and lasted for 12 h following extubation | **Postoperative usual care** (including chest physiotherapy, COT with venti-mask ) | 58 | NR | 178 | 136 | 11 (20) | This study had no clear definition of PPCs, but it presented data on the incidence of atelectasis. |
| Stock/1984 | Single-center (USA) | 38 | Cardiac surgery; Elective | | **CPAP** (supporting pressure: 7.5 cmH_2_O) was initiated at 2 h after extubation and lasted for 15 min every 2 h from the 2nd to 72nd h after extubation | **Postoperative usual care** (including deep breathing, incentive spirometry, COT) | 57 | NR | 285 | 273 | 7 (18) | This study had no clear definition of PPCs, but it presented data on the incidence of atelectasis. |
| Zarbock/2009 | Multicentre (Germany) | 468 | Cardiac surgery (CABG or heart valve replacement); Elective | | **CPAP** (supporting pressure: 10 cmH_2_O) via a facemask was applied immediately after extubation and lasted for at least 6 h following extubation | **Postoperative usual care** (including COT physiotherapy, intermittent CPAP for 10 min every 4 h) | 64 | 26.2 | 213 | 211 | NR | PPCs was defined as the composite of PaO_2_/FiO_2_ < 100, atelectasis, pneumonia, or reintubation. |
| Corley/2015 | Single-center (Australia) | 155 | Cardiac surgery (using CPB); Elective | | **HFNC** was delivered immediately after extubation to maintain SpO_2_ ≥ 95% for a minimum of 8 h with short breaks for nasal care or mobilisation | **Postoperative usual care** (including physiotherapy, early mobilisation, COT using nasal cannula or face mask to maintain  SpO_2_ ≥ 95%) | 64 | 35.5 | 96 | 104 | 23 (15) | This study had no clear definition of PPCs, but it presented data on postoperative ARF that required non-invasive or invasive mechanical ventilation. |
| Parke/2013 | Single-center (New Zealand) | 340 | Cardiac surgery (using CPB); Elective | | **HFNC** was delivered immediately after extubation to maintain SpO_2_ ≥ 93% until 9 a.m. on Day 2 after surgery | **Postoperative usual care** (including COT using simple facemask or nasal prongs to maintain SpO_2_ ≥ 93%) | 66 | 28.8 | NR | NR | NR | This study had no clear definition of PPCs, but it presented data on postoperative ARF that required non-invasive or invasive mechanical ventilation. |
| Pibul/2021 | Single-center (Thailand) | 67 | Cardiac surgery (using CPB); Elective | | **HFNC** was delivered immediately after extubation to maintain SpO_2_ ≥ 92% for 24 h | **Postoperative usual care** (including incentive spirometer and deep breathing exercises, COT using cannula or mask with a bag to maintain SpO_2_ ≥ 92%) | 62 | 25 | 144 | 130 | 15 (22) | This study had no clear definition of PPCs, but it presented data on pneumonia and postoperative ARF that required reintubation. |
| Sahin/2018 | Single-center (Turkey) | 100 | Cardiac surgery (CABG); Elective | | **HFNC** was delivered immediately after extubation to maintain SpO_2_ ≥ 93% | **Postoperative usual care** (including physiotherapy, COT using face mask to maintain SpO_2_ ≥ 93%) | 62 | 32.4 | 91 | 91 | 43 (43) | This study had no clear definition of PPCs, but it presented data on pneumonia and postoperative ARF that required non-invasive or invasive mechanical ventilation. |
| Tatsuishi/2020 | Single-center (Japan) | 148 | Cardiac surgery (CABG); Mixed (Elective 86%) | | **HFNC** was delivered immediately after extubation to maintain normal mixed venous oxygen saturation (60–80%) or to avoid a low SpO_2_ (< 90%) on the day of surgery and on postoperative day 1 in the ICU | **Postoperative usual care** (including COT using face mask to maintain SpO_2_ ≥ 90%) | 69 | NR | 173 | 182 | 89 (60) | No clear definition of PPCs, and no record of any composites of PPCs that defined by this NMA. |
| Zochios/2018 | Single-center (United Kingdom ) | 94 | Cardiac surgery (CABG, valve surgery or both); Elective | | **HFNC** was delivered after extubation to maintain SpO_2_ ≥ 95% for the first 24 h after surgery | **Postoperative usual care** (including COT using nasal prongs or facemask to maintain SpO_2_ ≥ 95% for the first 24 h after surgery) | 68 | 31.1 | 197 | 202 | 20 (21) | This study had no clear definition of PPCs, but it presented data on postoperative ARF that required non-invasive or invasive mechanical ventilation. |
| Abrard/2022 | Multicentre (French) | 253 | Cardiac or aortic surgery using CPB (more than 80% of all subjects); Elective or semi-urgent | | **NIV** with a mask was initiated as soon as possible after extubation and lasted for at least 6-8 h during the first 24 h after surgery. IPAP was started at 5-8 cm H_2_O and progressively increased by 2-3 cmH_2_O to obtain a tidal volume of 6-8 mL/kg and a RR less than 30/min; EPAP: 5-10 cmH_2_O. | **Postoperative usual care** (including chest physiotherapy, COT) | 67 | 26.8 | 213 | 222 | 32 (12.6) | This study had no clear definition of PPCs, but it presented data on all the composites of PPCs that defined by this NMA (including postoperative ARF, atelectasis, pneumonia, and pulmonary aspiration). |
| Al Jaaly/2013 | Single-center (United Kingdom) | 126 | Cardiac surgery (CABG using CPB); Elective | | **NIV** with an oronasal mask was initiated immediately after weaning and extubation, and was discontinued after 24 h. IPAP was 12 cm H_2_O and EPAP was 5 cm H_2_O, if BMI < 30; IPAP was 17 cmH_2_O and EPAP was 10 cmH_2_O, if BMI ≥ 30. | **Postoperative usual care** (including chest physiotherapy, coughing exercises, incentive spirometry, COT) | 68 | 28.5 | 132 | 115 | 59 (46.8) | PPCs was defined as the composite of postoperative ARF requiring CPAP or reintubation, atelectasis, pneumonia, and pleural effusion. |
| Celebi/2008 | Single-center (Turkey) | 100 | Cardiac surgery (CABG using CPB); Unknown | | **Arm 1:**  RM during IMV, **and NIV** with a facemask was initiated at 0.5 h after extubation and lasted 1 h every 6 h throughout the first 24 h. IPAP: was adjusted to about 10 cmH_2_O to achieve a tidal volume of 8 mL/kg; EPAP: 5 cmH_2_O.  **Arm 2:**  **NIV** with a facemask was initiated at 0.5 h after extubation and lasted 1 h every 6 h throughout the first 24 h. IPAP: was adjusted to about 10 cmH_2_O to achieve a tidal volume of 8 mL/kg; EPAP: 5 cmH_2_O. | **Arm 1:** RM during IMV, and **postoperative usual care** (including chest physiotherapy, incentive spirometry, COT).  **Arm 2: Postoperative usual care** (including chest physiotherapy, incentive spirometry, COT) | 56 | NR | NR | NR | NR | This study had no clear definition of PPCs, but it presented data on atelectasis. |
| Matte/2000 | Single-center (Belgium) | 96 | Cardiac surgery (CABG using CPB); Elective | | **Intervention 1**: **CPAP** (supporting pressure: 5 cmH_2_O) was started 4 h after extubation for 1 h every 3 h;  **Intervention 2**: **NIV** with an oronasal mask was started 4 h after extubation for 1 h every 3 h. IPAP: 12 cmH_2_O; EPAP: 5 cmH_2_O. | **Postoperative usual care** (including chest physiotherapy, incentive spirometry, COT) | 64 | 27.2 | NR | NR | NR | This study had no clear definition of PPCs, but it presented data on atelectasis. |
| Stéphan/2015 | Multicentre (France) | 830 | Cardiothoracic surgery (primarily CABG and valvular surgery); Unknown | | **NIV** was delivered with a full-face mask after extubation to maintain SpO_2_ at 92% to 98% and was used initially for 2 h and then for approximately 1 h every 4 h. IPAP: was started at 8 cmH_2_O and adjusted to achieve a tidal volume of 8 mL/kg and a RR less than 25/min; EPAP: 4 cmH_2_O. | **HFNC** was delivered continuously after extubation to maintain SpO_2_ between 92% and 98% | 64 | 28.3 | 137 | 137 | 152(18.3) | This study had no clear definition of PPCs, but it presented data on nosocomial pneumonia. |

Data on age and BMI are presented as mean ± standard deviation or median with interquartile range.

BMI body mass index; ECC extracorporeal circulation; PPCs postoperative pulmonary complications; CABG coronary artery bypass grafting; CPB cardiopulmonarybypass; COT conventional oxygenation therapy; ICU intensive care unit; RM recruitment maneuver; NR no record; NMA network meta-analysis;ARF acute respiratory failure; PaCO_2_ atrial partial pressure of carbon dioxide; FiO_2_ fraction of inspired oxygen; SpO_2_ oxygen saturation as measured by pulse oximetry; PaO2 arterial oxygen partial pressure; IPAP inspiratory positive airway pressure; EPAP expiratory positive airway pressure; RR respiratory rate.

# Table S2. Outcome measures in each included study.

| First author /Publication year | PPCs  (events/total) | | | Pneumonia (events/total) | | Atelectasis (events/total) | | Reintubation  (events/total) | | Short-term mortality  (death/total) | | Length of ICU stay (days) | | Length of in-hospital stay(days) | |
| --- | --- | --- | --- | --- | --- | --- | --- | --- | --- | --- | --- | --- | --- | --- | --- |
|  | EG | CG | | EG | CG | EG | CG | EG | CG | EG | CG | EG | CG | EG | CG |
| Al-Mutairi/2012 | - | - | | - | - | - | - | - | - | 3/72 | 0/36 | - | - | - | - |
| Jousela/1994 | 8/15 | 7/15 | | - | - | 8/15 | 7/15 | - | - | - | - | - | - | 13 ± 3 | 13 ± 2 |
| Matte/2000-*comparison1****** | 2/13 | 4/13 | | - | - | 2/13 | 4/13 | - | - | - | - | 50.1 ± 11.4 h | 53.2 ± 27.9 h | - | - |
| Pinilla/1990 | 30/32 | 21/26 | | - | - | 30/32 | 21/26 | - | - | - | - | 2.8 ± 1.1 | 2.7 ± 1.3 | 13 | 13 |
| Stock/1984 | 9/13 | 23/25 | | - | - | 9/13 | 23/25 | - | - | - | - | - | - | - | - |
| Zarbock/2009 | 12/232 | 25/236 | | 1/232 | 5/236 | - | - | - | - | - | - | 27 ± 1.6 h | 28 ± 1.7 h | 13 ± 0.5 | 14 ± 0.6 |
| Corley/2015 | 3/81 | 4/74 | | - | - |  | -- | 0/81 | 2/74 | - | - | 38.7 ± 35.2 h | 38.6 ± 23.9 h | - | - |
| Matte/2000-*comparison2****** | 2/13 | 6/19 | | - | - | 2/13 | 6/19 | - | - | - | - | 49.9 ± 14.5 h | 53.2 ± 27.9 h | - | - |
| Parke/2013 | 11/169 | 5/171 | | - | - | - | - | 2/169 | 2/171 | 1/169 | 1/171 | 33.4 ± 22.8 h | 28.9 ± 24 h | 11.6 ± 6.6 | 11.4 ± 6.7 |
| Pibul/2021^#^ | 4/34 | 1/33 | | 4/34 | 0/33 | - | - | 7/34 | 1/33 | - | - | 3 (2 - 5) | 3 (2 - 3) | 9 (7 - 12) | 8 (6 - 9) |
| Sahin/2018^#^ | 0/50 | 4/50 | | 0/50 | 2/50 | - | - | 0/50 | 4/50 | 0/50 | 2/50 | 2.4 ± 0.5 | 2.8 ± 1.7 | 6.5 ± 0.7 | 6.9 ± 1.1 |
| Tatsuishi/2020 | - | - | | - | - | - | - | - | - | - | - | 1 (1 - 1) | 1 (1 - 1) | 8 (7 - 10) | 9 (7 - 12) |
| Zochios/2018 | 3/49 | 6/45 | | - | - | - | - | 1/51 | 5/49 | 1/49 | 1/45 | 1 (1 – 2) | 1 (1 – 2) | 7 (6 - 9) | 9 (7 – 16) |
| Abrard/2022^#^ | 61/125 | 73/128 | | 3/125 | 3/128 | 61/125 | 73/128 | 9/125 | 8/128 | 0/125 | 0/128 | 4 (4 - 5) | 4 (4 - 5) | 4.3 ± 0.75 | 4.3 ± 0.75 |
| Al Jaaly/2013 | 9/63 | 23/63 | | 0/63 | 2/63 | 2/63 | 15/63 | 1/63 | 2/63 | 1/63 | 1/63 | 20 (17 - 40) h | 24 (18 - 42) h | 5 (4 - 6) | 6 (5 - 7) |
| Celebi/2008-arm1 | 10/25 | 15/25 | | - | - | 10/25 | 15/25 | - | - | - | - | 46 ± 3 h | 45 ± 7 h | 7.7 ± 1 | 7.3 ± 1.5 |
| Celebi/2008-arm2 | 12/25 | 23/25 | | - | - | 12/25 | 23/25 | - | - | - | - | 49 ± 9 h | 47 ± 9 h | 7.9 ± 1 | 7.5 ± 1 |
| Matte/2000-*comparison3****** | 3/19 | 3/19 | | - | - | 3/19 | 3/19 | - | - | - | - | 50.1 ± 11.4 h | 49.9 ± 14.5 h | - | - |
| Stéphan/2015 | 90/416 | | 83/414 | 90/416 | 83/414 | - | - | 57/416 | 58/414 | 23/418 | 28/414 | 6 (4 - 10) | 6 (4 - 10) | 14 (9 - 20) | 13 (9 - 23) |

Continuous data are presented as mean ± standard deviation or median with interquartile range.

EG experimental group; CG control group; PPCs postoperative pulmonary complications; ICU intensive care unit.

*****As Matte and colleagues simultaneously compared 3 methods in a single trial, we thus separate this study into three pairs of comparison (comparison 1: CPAP vs. COT; comparison 2: NIV vs. COT; comparison 3: CPAP vs. NIV). The number of event and total subjecs in each group are devided into 2 parts proportionally to form 3 pairs of comparison, guaranteing that the obsevered incidence of atelectasis in each group is unchanged.

**^#^**Study that had no explicit definition of PPCs but reported data on multiple composites of PPCs defined by this meta-analysis. In this case, we estimated the incidence of PPCs as the data on the component with a maximum event. Of note, the incidence of PPCs was estimated by using the data on the incidence of atelectasis in the study by Abrard et al., and using the incidence of postoperative actue respiratory failure requiring intubation in the study by Pibul et al. and the study by Sahin et al.

| **Incidence of PPCs;** RR (95% CI) | | | |
| --- | --- | --- | --- |
| **PUC** | 0.85 (0.60, 1.20) | 0.74 (0.46, 1.20) | 0.67 (0.49, 0.93) |
|  | **CPAP** | 0.88 (0.48, 1.60) | 0.80 (0.50, 1.26) |
|  |  | **HFNC** | 0.91 (0.56, 1.46) |
|  |  |  | **NIV** |
| **Incidence of** **atelectasis;** RR (95% CI) | | | |
| **PUC** | 0.95 (0.67, 1.34) | 0.49 (0.11, 2.20) | 0.65 (0.45, 0.93) |
|  | **CPAP** | 0.51 (0.11, 2.42) | 0.68 (0.43, 1.09) |
|  |  | **HFNC** | 1.33 (0.28, 6.27) |
|  |  |  | **NIV** |
| **Reintubation;** RR (95% CI) | | | |
| **PUC** |  | 0.74 (0.24, 2.27) | 0.82 (0.29, 2.34) |
|  | **CPAP** |  |  |
|  |  | **HFNC** | 1.11 (0.41, 3.01) |
|  |  |  | **NIV** |
| **Short-term mortality;** RR (95% CI) | | | |
| **PUC** | 3.55 (0.19, 66.89) | 0.77 (0.20, 2.92) | 0.64 (0.16, 2.52) |
|  | **CPAP** | 0.22 (0.01, 5.45) | 0.18 (0.01, 4.63) |
|  |  | **HFNC** | 0.84 (0.50, 1.42) |
|  |  |  | **NIV** |
| **Length of ICU stay;** MD (95% CI) | | | |
| **PUC** | -0.57(-1.17, 0.02) | -0.08 (-0.52, 0.37) | -0.01 (-0.56, 0.55) |
|  | **CPAP** | 0.50 (-0.23, 1.23) | 0.57 (-0.23, 1.37) |
|  |  | **HFNC** | 0.07 (-0.53, 0.67) |
|  |  |  | **NIV** |
| **Length of in-hospital stay**; MD (95% CI) | | | |
| **PUC** | -0.08 (-1.76, -0.16) | -0.47 (-1.11 0.16) | 0.0 (-0.56, 0.55) |
|  | **CPAP** | 0.33 (-0.83, 1.48) | 0.80 (-0.30, 1.90) |
|  |  | **HFNC** | 0.47 (-0.32, 1.26) |
|  |  |  | **NIV** |

# Table S3. League table for networks of various noninvasive respiratory support.

RR relative risk; MD mean difference; CI confidence intervals.

PPCs postoperative pulmonary complications; ICU intensive care unit; CPAP continuous positive airway pressure; NIV noninvasive ventilation; HFNC high flow nasal cannula; PUC postoperative usual care.

# Figure S1. Forest plot and network plot for the reintubation


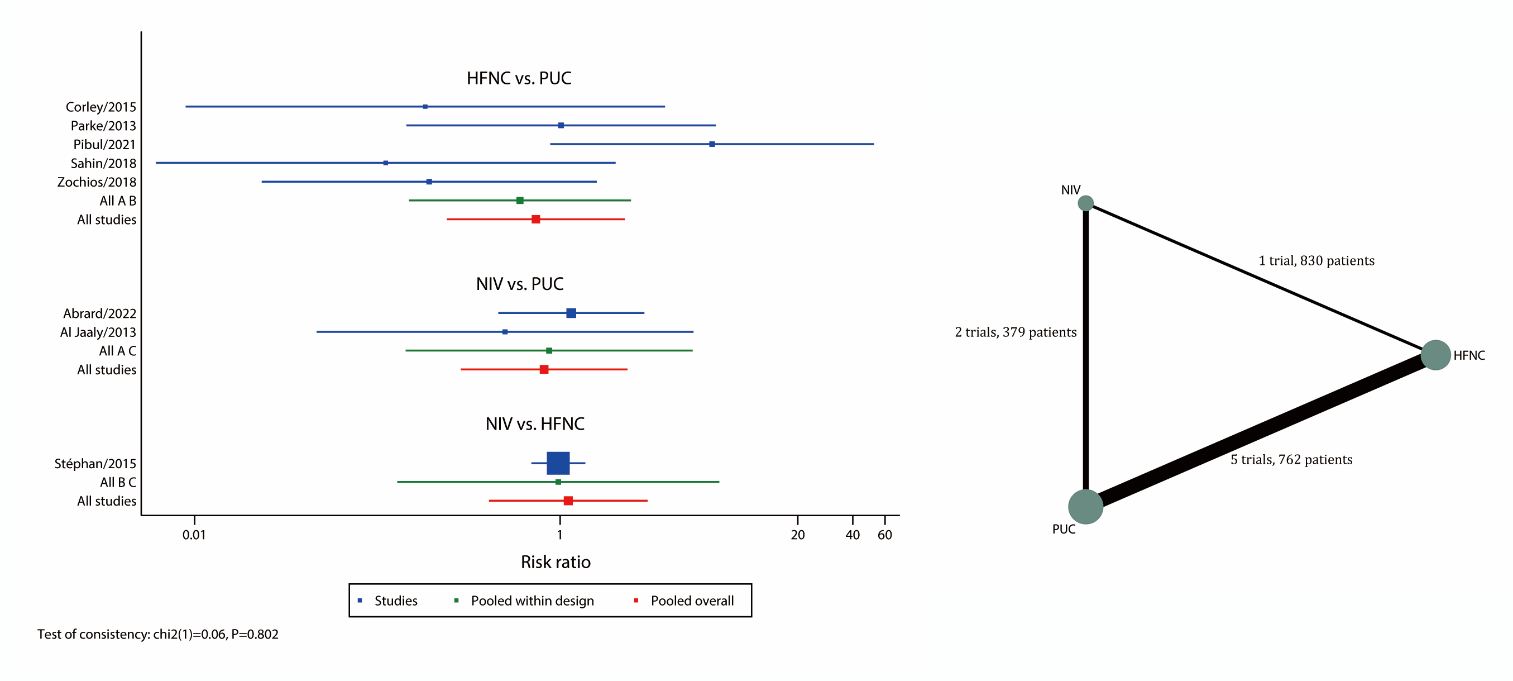


CPAP continuous positive airway pressure; NIV noninvasive ventilation; HFNC high-flow nasal cannula; PUC postoperative usual care.

A = PUC; B = HFNC; C = NIV

# Figure S2. Forest plot and network plot for the short-term mortality


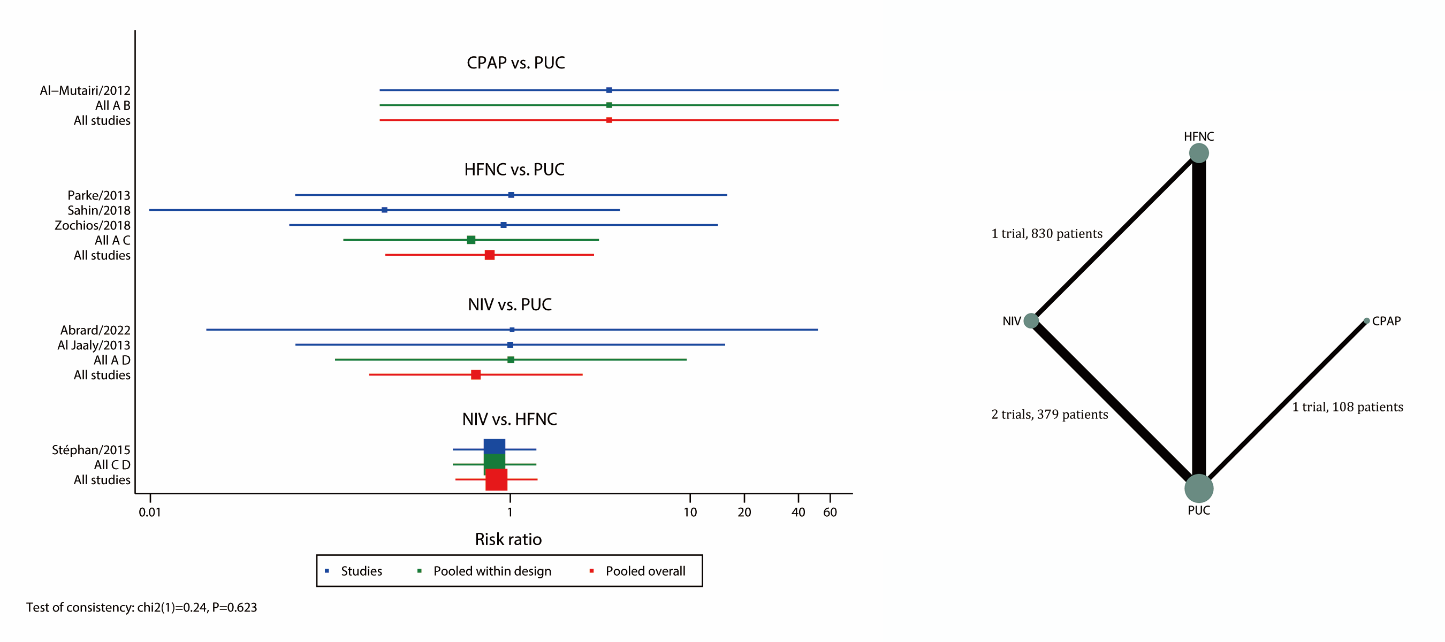


CPAP continuous positive airway pressure; NIV noninvasive ventilation; HFNC high-flow nasal cannula; PUC postoperative usual care.

A = PUC; B = CPAP; C = HFNC; D = NIV

# Figure S3. Forest plot and network plot for the length of ICU stay


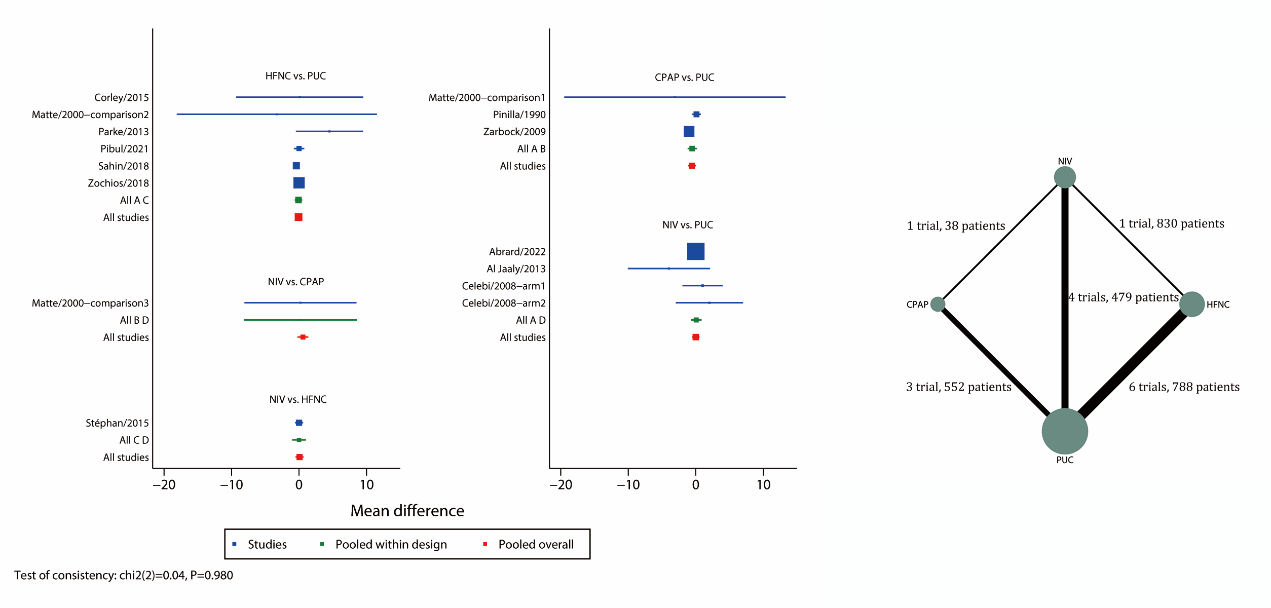


CPAP continuous positive airway pressure; NIV noninvasive ventilation; HFNC high-flow nasal cannula; PUC postoperative usual care.

A = PUC; B = CPAP; C = HFNC; D = NIV

# Figure S4. Forest plot and network plot for the length of in-hospital stay


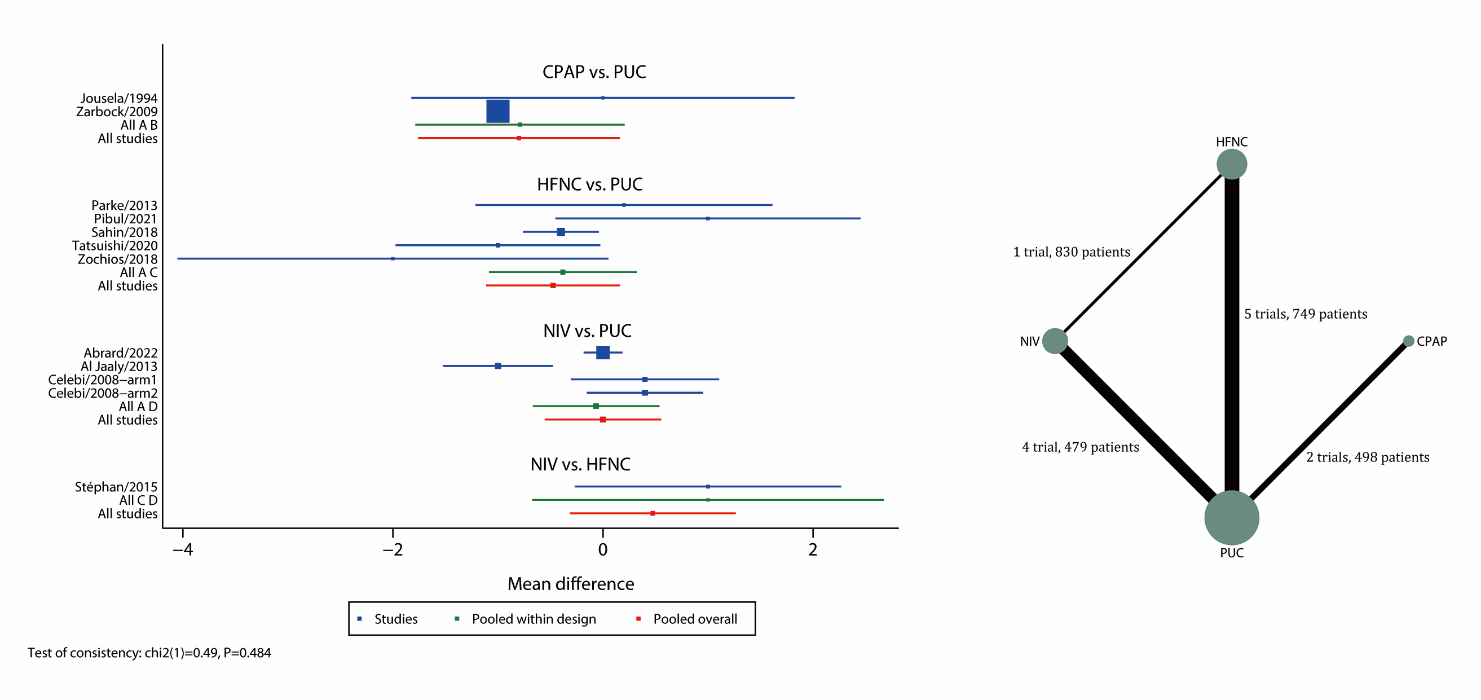


CPAP continuous positive airway pressure; NIV noninvasive ventilation; HFNC high-flow nasal cannula; PUC postoperative usual care.

A = PUC; B = CPAP; C = HFNC; D = NIV
